# A Longitudinal Study of the Association of Blood Unsaturated Fatty Acids With Posttraumatic Stress Disorder (PTSD)

**DOI:** 10.1002/npr2.12522

**Published:** 2025-02-17

**Authors:** Tomoko Inoue, Shintaro Ogawa, Zui Narita, Masayuki Sekiguchi, Yasushi Asari, Yuichi Kataoka, Jun Hattori, Hiroaki Hori, Yoshiharu Kim, Ken Inada

**Affiliations:** ^1^ Department of Psychiatry Kitasato University, School of Medicine Sagamihara Kanagawa Japan; ^2^ Department of Behavioral Medicine, National Institute of Mental Health National Center of Neurology and Psychiatry Tokyo Japan; ^3^ Department of Molecular Therapy, National Institute of Neuroscience National Center of Neurology and Psychiatry Tokyo Japan; ^4^ Department of Emergency and Critical Care Medicine Kitasato University School of Medicine Sagamihara Kanagawa Japan; ^5^ Emeritus Director General, National Institute of Mental Health National Center of Neurology and Psychiatry Tokyo Japan

**Keywords:** fatty acids, physical trauma, posttraumatic stress disorder, psychological symptoms, regression analysis

## Abstract

**Aim:**

This study aimed to investigate the association between blood fatty acid fractions and posttraumatic stress disorder (PTSD) judgment in individuals who have experienced physical trauma.

**Methods:**

Patients admitted to the emergency department for trauma, excluding those with brain damage or serious psychiatric disorders, were enrolled. Blood samples were collected on admission, and PTSD symptoms were assessed using a questionnaire 1 and 3 months after the injury. Multiple regression analysis was used to evaluate the association between fatty acids and Posttraumatic Diagnostic Scale severity scores, adjusting for age, sex, the Childhood Trauma Questionnaire (CTQ), and the use of psychotropic medications.

**Results:**

A significant association was observed between certain fatty acids and PTSD judgment. Mann‐Whitney U test results revealed that arachidonic acid was associated with PTSD judgment at 1 month and palmitic acid, stearic acid, oleic acid, linoleic acid, linolenic acid, eicosenoic acid, and eicosadiene acid with PTSD judgment at 3 months. Multiple regression analysis revealed that stearic acid, linoleic acid, arachidic acid, docosatetraenoic acid, lignoceric acid, docosahexaenoic acid, and total omega‐6 fatty acids (ω6) were associated with PTSD judgment after 1 month after trauma. In contrast, only linoleic acid and total ω6 were associated with PTSD judgment 3 months after trauma.

**Conclusions:**

This study is the first to enroll patients with general physical trauma and examine the relationship between fatty acids and PTSD. The findings suggest a potential relationship between blood fatty acid fractions and the development of PTSD symptoms in individuals who have experienced physical trauma. However, further research is needed to confirm and expand on these findings.

## Abstract 2

In this study, we analyzed the relationship between blood fatty acid fractions and PTSD in patients who were treated in the emergency room due to physical trauma. The results showed a relationship between the ω6 unsaturated fatty acids, especially linoleic acid, and PTSD judgment.

## Graphical abstract

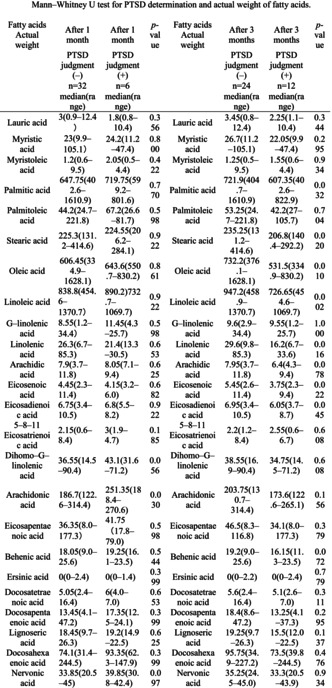

## 1 Introduction

Posttraumatic stress disorder (PTSD) is a mental disorder that can develop after experiencing a life‐threatening traumatic event. It manifests through a wide range of symptoms, including intrusive symptoms such as flashbacks, avoidance behaviors, pervasive negative emotional states, and hyperarousal [1]. Treatment options for PTSD include pharmacotherapy with selective serotonin reuptake inhibitors and psychotherapy with prolonged exposure therapy [2, 3]. However, neither treatment is effective for all patients, and psychotherapy can be time‐consuming and costly [3]. Furthermore, established prevention methods for PTSD are lacking, highlighting the urgent need for new and effective treatment and prevention approaches [4, 5].

Recently, the phospholipid hypothesis has been proposed, positing a correlation between the fatty acid composition of membrane phospholipids and neuropsychiatric function. Moreover, the relationships between blood unsaturated fatty acids and clinical symptoms of psychiatric disorders have been reported [6, 7]. For social anxiety, a strong negative correlation has been observed between clinical symptom rating scale scores and blood omega‐3 (ω3)‐unsaturated fatty acid levels [8]. In women with anxiety, a strong negative correlation has been noted between clinical symptom rating scale scores and docosahexaenoic acid (DHA) intake [9]. Notably, research has also explored the treatment of anxiety through external regulation of fatty acid intake [10, 11].

Unsaturated fatty acids are suggested to be involved in the pathogenesis of PTSD through multiple mechanisms, such as processes related to neuronal survival and plasticity, neurotransmitter/sympathetic activity, the hypothalamic–pituitary–adrenal system, inflammation, oxidative stress, and brain‐derived neurotrophic factor [12]. In addition, ω3‐unsaturated fatty acids are associated with volumetric changes in the hippocampus and amygdala [13], with a reported tendency for reduced hippocampal volume in individuals with PTSD [14]. The enhancement of hippocampal neurogenesis also plays an important role in the process underlying fear extinction, promoting the attenuation of conditioned fear memories [15]. Therefore, these fatty acids may be closely involved in the pathogenesis of PTSD through the hippocampal axis.

In a study comparing erythrocyte membrane fatty acid concentrations between patients with PTSD and healthy controls, DHA concentrations were significantly lower in the patient group [16]. In animal experiments, adjusting the ratio of ω3 to ω6‐unsaturated fatty acids in the diet markedly reduces conditioned fear memory following traumatic experiences [17]. In humans, ω3‐unsaturated fatty acids can alleviate PTSD and anxiety symptoms and mitigate the development of PTSD symptoms after a car accident [18, 19, 20]. However, the effects may not be sufficient. It is therefore suggested that there is a specific relationship between anxiety, including PTSD, and unsaturated fatty acids, indicating that adjusting the amount of unsaturated fatty acids could help mitigate the onset of PTSD. However, existing research on the association between PTSD symptoms and unsaturated fatty acids in humans is limited, particularly regarding PTSD symptoms that occur following physical traumatic experiences.

In the present study, we hypothesized that there is an association between PTSD judgment and blood fatty acid fractions in trauma survivors. Therefore, to test the hypothesis, we aimed to analyze the association between 24 fractions of fatty acids, including the ratio of ω3 and ω6 fatty acids, in the blood and PTSD judgment at one and 3 months following injury in patients admitted to the emergency department owing to physical trauma.

## 2 Methods

### 2.1 Study Design and Participants

This prospective case–control study was conducted at a single institution, enrolling consecutive patients admitted to Kitasato University Hospital Emergency Center for trauma via emergency transport from August 2019 to March 2023. Cases were collected for a total of 286 days from (1) August 9 to August 26, 2019; (2) July 15 to October 20, 2021; (3) May 16 to July 19, 2022; (4) December 1 to December 22, 2022; and (5) January 7 to March 30, 2023. Participants aged between 18 and 80 years old were enrolled. However, those aged 70 and over were required to recite at least four digits backward in the Digit Span task. The exclusion criteria included: (1) individuals with organic brain parenchymal disorder identified on brain imaging; (2) those undergoing treatment for schizophrenia, mood disorder, epilepsy, neurodegenerative disease, or other serious mental disorder before the injury that necessitated the emergency transport; (3) those whose mental or physical condition was seriously compromised, preventing them from tolerating the survey; and (4) those whose native language was not Japanese.

### 2.2 Research Methods and Evaluation Items

#### 2.2.1 Evaluation of Psychiatric Symptoms

Patients who had completed initial physical treatment and were deemed interviewable by their primary physicians were informed about the study by the researcher. Those who provided consent were interviewed by the psychiatrist in charge of the study, who inquired about their current psychiatric symptoms, history of mental illness, and treatment history to ensure they met the study selection criteria. The Childhood Trauma Questionnaire (CTQ) [21] was also used to assess childhood trauma. The following three questionnaires were administered 1 and 3 months after the injury to assess psychiatric symptoms.

1. Posttraumatic Diagnostic Scale (PDS): sassed on the Diagnostic and Statistical Manual of Mental Disorders (4th edition; DSM‐IV) [22], the PDS is a self‐administered scale for assessing severity and diagnosing PTSD. Parts 1 and 2 inquire about the traumatic event and allow free‐response statements. Part 3 evaluates re‐experiencing, avoidance, and hyperarousal on a 4‐point scale corresponding to the DSM‐IV criteria [1], while Part 4 assesses impairment in daily functioning.
2. Hospital Anxiety and Depression Scale (HADS): [23] This scale measures anxiety and depression over the past week and consists of 14 items rated on a 4‐point scale.
3. World Health Organization Quality of Life: [24] The WHO‐QOL26 24A is a self‐administered 5‐point scale consisting of 26 items that assess the quality of life during the past two weeks.

Questionnaires were collected in person for individuals who were hospitalized and by mail for those who had already been discharged.

#### 2.2.2 Assessment of Blood Fatty Acids

Blood samples collected for the treatment of related physical diseases at the time of hospitalization, for which consent was obtained, were used for analysis in this study. Blood fatty acid fractions (24 components) were measured using gas chromatography at an external laboratory (SRL Inc., Tokyo) using a gas chromatography (Shimadzu Science East Corporation, Tokyo, Japan).

#### 2.2.3 Other Endpoints

Age, sex, body mass index, medications administered during hospitalization, and blood test results were obtained from medical records. The Injury Severity Score (ISS), which evaluates the severity of trauma, was obtained from the Trauma Registry of Kitasato University Hospital Emergency Center.

### 2.3 Statistical Analysis

We analyzed the relationship between the 24 fractions of fatty acids in the blood at admission and PTSD symptoms at 1 and 3 months after injury. The presence of PTSD symptoms and PTSD determination were assessed using the PDS‐4. The Mann–Whitney U test was performed for PTSD determination using PDS‐4 and fatty acid fractionation. Multiple regression analysis was used to evaluate the association between fatty acids and PDS severity scores after adjusting for age, sex, CTQ, and psychotropic medication use. In addition, a permutation test was performed to address type I errors. Random forest imputation was used to address missing data.

Statistical analysis was performed using IBM SPSS Statistics Standard Authorized User Version 29 (IBM, Armonk, NY, USA) for the Mann–Whitney U test. R software Version 4.4.1 (R Foundation, Vienna, Austria) was used for multiple regression analysis employing permutation tests and random forest imputation, as SPSS does not support these programs. A *p*‐value < 0.05 was set as the significance level.

For the Mann–Whitney U test and other exploratory studies, multiple testing correction was not performed. However, for the main results, *p*‐values were corrected using a permutation test.

### 2.4 Ethical Considerations

This study was conducted in accordance with the Declaration of Helsinki and was approved by the Ethics Committee of Kitasato University School of Medicine and Hospital (approval number: C19‐054). This study also received ethics approval (approval number: B2023‐040) from the National Center of Neurology and Psychiatry, the National Institute of Neurology and Psychiatry, which is a research sub‐center, and the Kitasato University School of Medicine and Hospital Ethics Committee in a collective review. All participants were fully informed about the study design and provided written consent.

## 3 Results

### 3.1 Patient Enrollment and Classification

**FIGURE 1:**
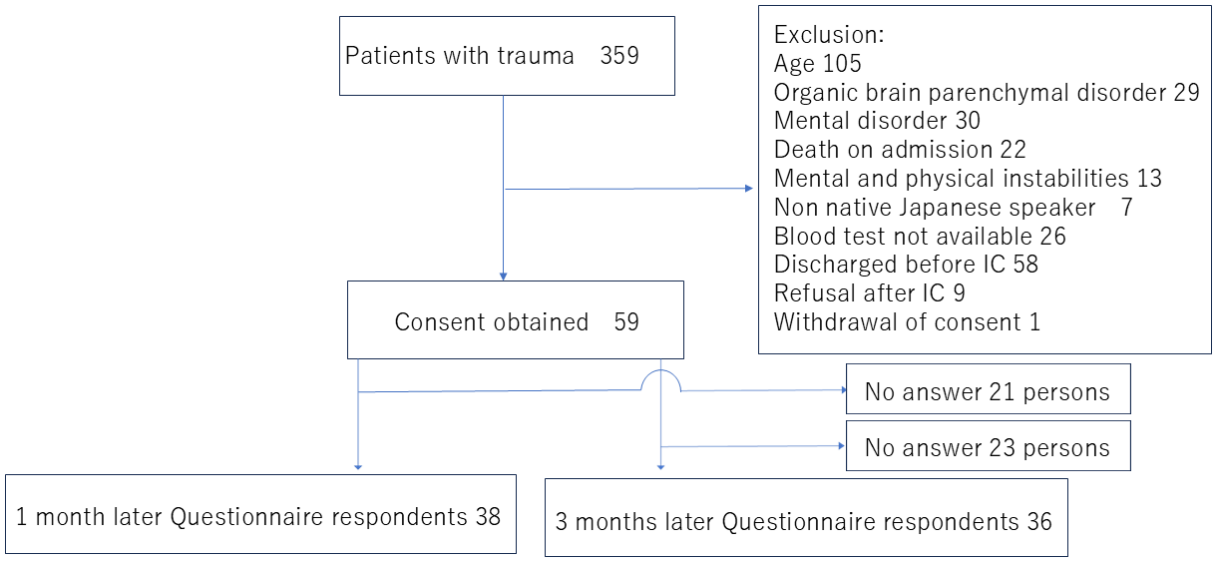
Enrollment flow chart of this study.

During the 286 days of case collection, 359 patients were admitted to the Rescue Center owing to trauma. Among them, 153 patients met the selection criteria and did not meet any exclusion criteria. Of these 153 patients, 127 patients had sufficient blood samples available for fatty acid determination. Of these, 69 patients were informed about the study and were invited to participate, and 60 provided informed consent. However, one patient withdrew consent at the time of answering the questionnaire and discontinued participation in the study. Of the remaining 59 study participants, questionnaires were collected from 38 patients 1 month after the injury, resulting in a collection rate of 64.4%. Questionnaires were collected from 36 participants 3 months after the injury, representing a collection rate of 61.0% (Figure 1). Questionnaires were collected at both 1 and 3 months after the injury from 35 participants (59.3% response rate). Three participants answered the questionnaires at 1 month only, while two completed the questionnaire at 3 months only; one of these two participants had many missing values, preventing the assessment of PTSD determination and severity. Thus, the total number of participants included for analysis in this study was 39. The characteristics of these participants are presented in Table 1. Overall, 39 participants completed the questionnaire evaluation after 1 or 3 months, with a median age of 57 years and 69.2% male participants.

**TABLE 1:** Demographic, clinical, and psychosocial characteristics of participants at the baseline assessment.

Characteristics	*N*	%
Number of individuals	39	
Age, median	57 (18–79)	
Sex, male	27	69.2
BMI, mean ± SD	24.2 ± 4.1	
BMI, median	24.6 (15.1–34.2)	
ISS, median	4 (1–29)	
ISS Mean ± SD	7.8 ± 7.2	
PTSD severity assessed via at 1 month (*N* = 38) mean ± SD	10.6 ± 10.4	
PTSD severity assessed via PDS at 3 months (*N* = 36) mean ± SD	9.4 ± 9.8	
CTQ None of the above	14	35.9
Psychotropic medication	29	74.4
Hyperlipidemic medication	0	0

Abbreviations: BMI, body mass index; CTQ, childhood trauma questionnaire; ISS, injury severity score; PTSD, posttraumatic stress disorder.

An ISS of 15 or higher is classified as severe or potentially severe [25]. However, the participants had a mild ISS, with a median score of 4 and a mean score of 7.8. Fourteen participants (35.9%) did not meet any of the childhood trauma items on the CTQ, whereas 29 (74.4%) received psychotropic medication during hospitalization. None of the patients received hyperlipidemic medication during hospitalization. Based on PDS assessments at 1 and 3 months, participants who met the diagnostic criteria for PTSD were classified in the group with PTSD determination, while those who did not meet the criteria were classified in the group without PTSD determination.

### 3.2 Characteristics of Patients at 1 and 3 Months After Injury

The characteristics of patients at 1 and 3 months are presented in Table 2 for the groups with and without PTSD determination. Of the 38 patients with valid responses after 1 month, six were classified in the group with PTSD determination. Among the 36 patients with valid responses after 3 months, 12 were classified in the group with PTSD determination. Notably, the median age tended to be lower in the groups with PTSD determination at both 1 and 3 months than in those without PTSD determination.

**TABLE 2:** Demographic, clinical, and psychosocial characteristics of participants at 1 and 3 months post‐injury.

	Questionnaire collection after 1 month	PTSD after 1 month	PTSD after 1 month	*p*	Questionnaire collection after 3 months	PTSD after 3 months	PTSD after 3 months	*p*
	Total	(+)	(−)		Total	(+)	(−)	
	Median (range)	Median (range)	Median (range)		Median (range)	Median (range)	Median (range)	
Number of individuals	38	6	32		36	12	24	
Age	56 (18–79)	39 (20–77)	57.5 (18–79)	0.316	57.5 (18–79)	54.5 (18–70)	62 (20–79)	0.078
Sex, Male (%)	27 (71.1)	2 (33.3)	25 (78.1)	0.470	24 (66.7)	6 (50)	18 (75)	0.157
BMI at admission	24.6 (15.1–32.4)	23.2 (21.9–25.7)	24.7 (15.1–32.4)	0.770	24.6 (15.1–34.2)	25.5 (15.1–34.2)	24.6 (18.0–32.4)	0.456
ISS	4 (1–29)	4 (1–9)	4.5 (1–29)	0.245	4 (1–29)	9 (1–25)	4 (1–29)	0.045
Psychotropic medication (%)	28 (73.7)	4 (66.7)	24 (75)	0.644	26 (72.2)	10 (83.3)	16 (66.7)	0.438
CTQ_Total	33 (25–71)	27.5 (25–37)	33.5 (25–71)	0.082	33 (25–86)	36.5 (27–86)	31 (25–49)	0.057
PDS_1M_PTSD Severity	10 (0–48)	20 (13–23)	7 (0–48)	0.003	9 (0–48)	16 (7–48)	2.5 (0–23)	0.000
PDS_3M_PTSD Severity	6 (0–33)	14 (0–21)	4 (0–33)	0.272	6.5 (0–33)	21 (9–33)	2.5 (0–14)	0.000
HADS_1M_T	11.5 (0–31)	14.5 (5–24)	10 (0–31)	0.199	12 (0–31)	16 (1–31)	6 (0–22)	0.001
HADS_3M_T	7 (0–30)	9 (3–26)	7 (0–30)	0.654	8 (0–30)	18 (9–30)	6 (0–20)	0.000
WHOQOL26_1M_QOL_Average	3.2 (2.1–4.3)	3.1 (2.8–3.7)	3.2 (2.1–4.3)	0.830	3.1 (2.1–4.3)	2.8 (2.1–3.2)	3.4 (2.5–4.3)	0.002
WHOQOL26_3M_QOL_Average	3.2 (2.2–4.7)	3.4 (2.3–3.9)	3.1 (2.2–4.7)	0.564	3.2 (2.2–4.7)	2.7 (2.3–3.4)	3.4 (2.2–4.7)	0.000

Abbreviations: BMI, body mass index; CTQ, childhood trauma questionnaire; ISS, injury severity score; PTSD, posttraumatic stress disorder.

The ISS was not significantly different between the groups, with a median score of 4 in the group with PTSD determination at 1 month and 4.5 in the group without PTSD determination at 1 month. However, at 3 months, the ISS was higher in the group with PTSD, with a score of 9 in the group with PTSD determination compared to a score of 4 in the group without PTSD (*p* < 0.05).

The mean PTSD severity after 1 month was higher in the group with PTSD than in the group without PTSD at both 1 and 3 months (*p* < 0.05). However, at 3 months, the mean PTSD severity was higher in the group with PTSD than in the group without PTSD (*p* < 0.05).

The total HADS was significantly higher in the group with PTSD determination at 3 months than in the group without PTSD at both 1 and 3 months (*p* < 0.05). However, the mean quality of life measured using the WHO‐QOL26 was significantly low in the group with PTSD determination after 3 months (*p* < 0.05).

### 3.3 Association Between Fatty Acids and PTSD

The results of the Mann–Whitney U test for the presence of PTSD judgment and actual fatty acid weight are depicted in Table 3. Significant differences (*p* < 0.05) were observed between the groups with and without PTSD judgment in the following parameters: determination after 1 month and arachidonic acid, and determination after 3 months and the actual weights of palmitic, stearic, oleic, linoleic, linolenic, eicosenoic, and eicosadiene acids. When unsaturated fatty acids were categorized into the ω3, ω6, and ω9 series for examination (Table S1), a significant difference was observed in the total actual weight of ω6 and ω9 unsaturated fatty acids between the groups with and without PTSD determination at 3 months (*p* < 0.05).

**TABLE 3:** Mann–Whitney U test for PTSD determination and actual weight of fatty acids.

Fatty acids actual weight	After 1 month	After 1 month	*p*	Fatty acids actual weight	After 3 months	After 3 months	*p*
	PTSD judgment (−)	PTSD judgment (+)			PTSD judgment (−)	PTSD judgment (+)	
	*n* = 32	*n* = 6			*n* = 24	*n* = 12	
	Median (range)	Median (range)			Median (range)	Median (range)	
Lauric acid	3 (0.9–12.4)	1.8 (0.8–10.4)	0.356	Lauric acid	3.45 (0.8–12.4)	2.25 (1.1–10.4)	0.344
Myristic acid	23 (9.9–105.1)	24.2 (11.2–47.4)	0.800	Myristic acid	26.7 (11.2–105.1)	22.05 (9.9–47.4)	0.295
Myristoleic acid	1.2 (0.6–9.5)	2.05 (0.5–4.4)	0.422	Myristoleic acid	1.25 (0.5–9.5)	1.55 (0.6–4.4)	0.934
Palmitic acid	647.75 (402.6–1610.9)	719.75 (599.2–801.6)	0.770	Palmitic acid	721.9 (404.7–1610.9)	607.35 (402.6–822.9)	0.032
Palmitoleic acid	44.2 (24.7–221.8)	67.2 (26.6–81.7)	0.598	Palmitoleic acid	53.25 (24.7–221.8)	42.2 (27–105.7)	0.704
Stearic acid	225.3 (131.2–414.6)	224.55 (206.2–284.1)	0.922	Stearic acid	235.25 (131.2–414.6)	206.8 (140.4–292.2)	0.020
Oleic acid	606.45 (334.9–1628.1)	643.6 (550.7–830.2)	0.861	Oleic acid	732.2 (376.1–1628.1)	531.5 (334.9–830.2)	0.010
Linoleic acid	838.8 (454.6–1370.7)	890.2)732.7–1069.7)	0.922	Linoleic acid	947.2 (458.9–1370.7)	726.65 (454.6–1069.7)	0.002
G–linolenic acid	8.55 (1.2–34.4)	11.45 (4.3–25.7)	0.598	G–linolenic acid	9.6 (2.9–34.4)	9.55 (1.2–25.7)	1.000
Linolenic acid	26.3 (6.7–85.3)	21.4 (13.3–30.5)	0.653	Linolenic acid	29.6 (9.8–85.3)	16.2 (6.7–33.6)	0.016
Arachidic acid	7.9 (3.7–11.8)	8.05 (7.1–9.4)	0.625	Arachidic acid	7.95 (3.7–11.8)	6.4 (4.3–9.4)	0.078
Eicosenoic acid	4.45 (2.3–11.4)	4.15 (3.2–6.0)	0.682	Eicosenoic acid	5.45 (2.6–11.4)	3.75 (2.3–9.4)	0.022
Eicosadienoic acid	6.75 (3.4–10.5)	6.8 (5.5–8.2)	0.922	Eicosadienoic acid	6.95 (3.4–10.5)	6.05 (3.7–8.7)	0.045
5–8–11 Eicosatrienoic acid	2.15 (0.6–8.4)	3 (1.9–4.7)	0.185	5–8–11 Eicosatrienoic acid	2.2 (1.2–8.4)	2.55 (0.6–6.7)	0.608
Dihomo–G–linolenic acid	36.55 (14.5–90.4)	43.1 (31.6–71.2)	0.056	Dihomo–G–linolenic acid	38.55 (16.9–90.4)	34.75 (14.5–71.2)	0.608
Arachidonic acid	186.7 (122.6–314.4)	251.35 (188.4–270.6)	0.030	Arachidonic acid	203.75 (130.7–314.4)	173.6 (122.6–265.1)	0.156
Eicosapentaenoic acid	36.35 (8.0–177.3)	41.75 (17.8–79.0)	0.598	Eicosapentaenoic acid	46.5 (8.3–116.8)	34.1 (8.0–177.3)	0.379
Behenic acid	18.05 (9.0–25.6)	19.25 (16.1–23.5)	0.544	Behenic acid	19.2 (9.0–25.6)	16.15 (11.3–23.5)	0.072
Ersinic acid	0 (0–2.4)	0 (0–1.4)	0.399	Ersinic acid	0 (0–2.2)	0 (0–2.4)	0.779
Docosatetraenoic acid	5.05 (2.4–16.4)	6 (4.0–7.0)	0.653	Docosatetraenoic acid	5.6 (2.4–16.4)	5.1 (2.6–7.0)	0.311
Docosapentaenoic acid	13.45 (4.1–47.2)	17.3 5 (12.5–24.1)	0.399	Docosapentaenoic acid	18.4 (8.6–47.2)	13.25 (4.1–37.3)	0.295
Lignoseric acid	18.45 (9.7–26.3)	19.2 (14.9–22.5)	0.625	Lignoseric acid	19.25 (9.7–26.3)	15.5 (12.0–22.5)	0.137
Docosahexaenoic acid	74.1 (31.4–244.5)	93.35 (62.3–147.9)	0.399	Docosahexaenoic acid	95.75 (34.9–227.2)	73.5 (39.8–244.5)	0.476
Nervonic acid	33.85 (20.5–45)	39.85 (30.8–42.4)	0.097	Nervonic acid	35.25 (24.5–45.0)	33.3 (20.5–43.9)	0.934

The Mann–Whitney U test examining the presence of PTSD determination and fatty acid percentage weights revealed significant differences in PTSD determination and dihomo‐gamma‐linolenic acid (%) after 1 month, as well as in PTSD determination and linolenic acid (%) and nervonic acid (%) after 3 months (*p* < 0.05) (Table S2).

Multiple regression analysis was performed to identify fatty acids associated with PTSD judgment after controlling for the effects of age, sex, CTQ, and psychotropic medication status. A permutation test was also performed to further address type I errors. The results demonstrated that stearic acid, linoleic acid, arachidic acid, behenic acid, docosaetetraenoic acid, lignoceric acid, docosahexaenoic acid (%), nervonic acid (%), total ω6, and total polyunsaturated fatty acids were associated with PTSD judgment 1 month after initial trauma (Table 4a). Linoleic acid and totalω6 were also associated with PTSD judgment 3 months after trauma (Table 4b).

**TABLE 4:** Multiple regression analysis, adjusted for age, sex, CTQ, and use of psychotropic drugs, and permutation test.

(a) After 1 month			
Fatty acid	Coefficient	*p*	*p*_permutation
Stearic acid	−0.062	0.006	0.038
Linoleic acid	−0.020	0.001	0.007
Arachidic acid	−2.051	0.005	0.025
Behenic acid	−0.984	0.003	0.025
Docosatetraenoic acid	−1.493	0.006	0.029
Lignoseric acid	−1.035	0.005	0.034
Docosahexaenoic acid (%)	3.194	0.009	0.034
Nervonic acid (%)	12.519	0.013	0.043
Total omega‐6	−0.016	0.001	0.010
Total PUFA	−0.011	0.012	0.033

(b) After 3 months			
	Coefficient	*p*	*p*_permutation
Linoleic acid	−0.01	0.02	0.05
Omega6 total	−0.01	0.01	0.04

Abbreviation: PUFA, polyunsaturated fatty acid.

Spearman's correlation coefficients were calculated for the blood sample results and linoleic acid levels at the initial visit, and those that were significant (*p* < 0.05) are presented in Table S3. In addition to total cholesterol, low‐density lipoprotein cholesterol, and triglycerides, correlations were observed with coagulation‐fibrinolytic parameters, including prothrombin percentage, prothrombin time, and fibrinogen, and indices of inflammation, such as C‐reactive protein levels, white blood cell count, and neutrophil count.

## 4 Discussion

In the present study, we investigated the relationship between fatty acid fractionation and the presence or absence of PTSD judgment in patients with physical trauma who presented to the emergency department. To the best of our knowledge, no other study has longitudinally examined this association in a sample of patients with general physical trauma. Notably, our study is the first to investigate this relationship in such a population.

In our study, 12 of 36 patients (33.3%) 3 month after injury had PTSD; however, this result should be interpreted cautiously. Matsuoka et al. reported that 31% of patients who experience a motor vehicle accident develop some form of psychiatric disorder, with 8% developing PTSD, and 16% exhibiting partial PTSD [26]. Therefore, the high rate of PTSD observed in this single‐center study may be attributed to patient selection bias.

The prevalence of PTSD in the general population is known to be about twice the risk for women as for men [27]. In this study, the proportion of males in the group with PTSD 3  months after injury was 50%. This may be because many trauma patients brought to the Kitasato University Hospital Emergency Center are male, and of the 39 subjects who agreed to participate in the study and responded to the questionnaire, 69.2% (27 subjects) were male. It is also known that there are gender differences in lipid metabolism [28]. Therefore, further investigation of potential gender‐specific influences on PTSD risk, including lipid metabolism, is warranted with a larger sample size. In addition, only a small proportion of participants in the present study met the life‐threatening DSM‐IV diagnostic criteria A2. In the PDS‐4 used in this study, PTSD criteria are considered met if participants answer “yes” to at least one of four items in Criterion A (injury or life‐threatening). As all the participants experienced trauma, they answered “yes” to the first of the four questions—“Were you injured?” Consequently, even if they answered “no” to the subsequent questions—“Did you think your life was in danger?” and “Did you think someone else's life was in danger”—the Criterion A requirement was still fulfilled. If the other criteria are also met, the patient may be classified as having PTSD. Therefore, a PTSD determination on the PDS does not necessarily equate to a formal diagnosis of PTSD. However, it is assumed that patients may be confused immediately after an injury, making it difficult for them to accurately answer whether they felt a threat to their lives. Moreover, as the A2 criterion in DSM‐IV in DSM‐5 was abolished owing to its unreliability, the present study was conducted under the assumption that the A2 item was not satisfied. Therefore, PTSD was considered in this study even if the A2 items were not met.

In terms of the relationship between fatty acids and PTSD as assessed by the PDS, linoleic acid (ω6) and total ω6 were higher in the group without PTSD than in the group with PTSD after 3 months. Moreover, the group of patients with PTSD determination had lower levels of ω6 fatty acids and linoleic acid. This result is consistent with previous studies demonstrating an association of linoleic acid with depression and PTSD [29, 30].

In the present study, we observed an association between the presence of posttraumatic PTSD and blood linoleic acid levels at the time of injury. Notably, assuming that blood fatty acid levels at the time of injury reflect the risk of developing PTSD, low linoleic acid levels at the time of injury are risk factors for developing PTSD in cases of physical trauma. Therefore, these results suggest that maintaining adequate amounts of linoleic acid and other unsaturated fatty acids may protect against PTSD. Although low blood linoleic acid levels have been reported in patients with depression [31], it remains controversial whether excessive linoleic acid intake is a risk for the development of psychiatric disorders [29, 30].

Blood linoleic acid levels are affected by diet. Westernized diets are generally high in polyunsaturated fatty acids, comprising 80%–90% of these fats, including linoleic acid. In addition, during physical invasion, fatty acid synthesis is regulated by hormones such as adrenaline and insulin [32, 33]. These diverse factors may account for the variability observed across studies. For example, studies, including ours, that examine PTSD and linoleic acid, typically measure linoleic acid after the event that triggered the onset of PTSD; however, baseline data in daily life may also be important [10]. In animal experiments using a PTSD model, adjusting the ω3/6 ratio through diet reduced PTSD‐like behaviors [17]. Therefore, linoleic acid should also be considered in terms of overall fatty acid composition, including the ω3/6 ratio, as well as its individual contribution (actual weight).

In this study, we only evaluated unsaturated fatty acids in blood at the time of admission immediately after injury. Therefore, it is unclear whether the low blood concentration of unsaturated fatty acids is a cause or a consequence of the development of PTSD in a causal relationship, we could only analyze how fatty acid composition at the time of injury affects subsequent psychiatric symptoms. Fatty acids in human blood fluctuate dynamically after physical trauma injury [34]. Therefore, their involvement in the onset of psychiatric symptoms, including PTSD, is assumed to also vary over time. Although trauma‐induced changes in lipid metabolism may influence PTSD symptoms, and a temporal relationship between trauma, lipid changes, and psychiatric outcome may occur, a causal relationship is not known because fatty acid composition was assessed only once in this study. For further validation, fatty acid assessment should not only be performed immediately after injury but at multiple time points thereafter.

In addition, unlike previous studies that have repeatedly noted an association between ω3‐unsaturated fatty acids and psychiatric disorders [8, 9, 35], we did not observe such an association with PTSD. This discrepancy could be attributed to various factors, including the dietary culture of participants, the presence or absence of physical trauma, and the method used to measure fatty acids in the blood. In addition, a study examining whether post‐injury DHA supplementation could prevent PTSD in patients with physical trauma found that it did not prevent PTSD symptoms at 3 months post‐injury [36]. This result is consistent with our finding of no association between ω3‐unsaturated fatty acids and the presence of a PTSD determination at 3 months. Although the present study and the previous study both involve participants with “physical trauma,” they differ in sample size, severity of physical trauma, and timing of the initial interview. In the present study, the sample size was small, with a median ISS of 4 and relatively minor injuries. Moreover, the timing of intervention initiation was late, occurring after the patient was discharged from the ICU. This selection bias may have affected the results of our study. Future research should investigate the relationship between fatty acids and both physical and psychological assessments, such as the presence and severity of physical trauma and the presence and severity of PTSD diagnoses.

In the present study, regarding the correlation between blood results and linoleic acid levels at the time of examination, we observed an association with the coagulation‐fibrinolytic system and indices of inflammation. Consistent with these findings, previous studies have demonstrated an association between psychiatric disorders and the coagulation‐fibrinolytic system [36, 37, 38], warranting further investigations. Although this study did not provide a definitive conclusion, a detailed examination of blood fatty acid fractions and PTSD symptoms may help prevent the subsequent progression to PTSD by regulating blood fatty acids through diet in various patients transported to emergency rooms and victims of disasters. Currently, there are no established preventive methods for PTSD; however, further research on blood fatty acids may contribute to the development of a preventive strategy that can be easily and effectively implemented for a large number of individuals.

This study has some limitations. First, this is a single‐center study, and the number of cases was limited. The sample size used for the regression analysis in this study was 36, a small sample size to obtain reliable regression analysis results. The circumstances and degree of injury events varied, and only a few were life‐threatening. Moreover, the extent of the injuries and the degree of trauma varied greatly among individuals. Second, the fatty acid composition was assessed once at the time of injury. As fatty acid composition changes depending on the trauma itself and post‐injury conditions, including diet, it is necessary to establish multiple evaluation points and examine the effects of nutritional content and fatty acid intake after hospitalization in the future. Therefore, a comprehensive study with a larger number of cases is needed.

## 5 Conclusion

In the present study, the analysis of the association between blood fatty acid fractions and PTSD judgment at 1 and 3 months after injury in patients who were transported to the emergency department owing to trauma revealed an association between PTSD judgment and ω6 unsaturated fatty acids, especially linoleic acid actual weight.

## Author Contributions

Kim Yoshiharu designed the study, the main conceptual ideas, and the proof outline. Tomoko Inoue, Yuuichi Kataoka, and Jun Hattori collected the data. Shintaro Ogawa and Ken Inada aided in interpreting the results and edited the manuscript. Yasushi Asari supervised the project. Tomoko Inoue wrote the manuscript with support from Shintaro Ogawa, Kim Yoshiharu, and Ken Inada. All authors discussed the results and commented on the manuscript.

## Ethics Statement

This study was conducted in accordance with the Declaration of Helsinki and was approved by the Ethics Committee of Kitasato University School of Medicine and Hospital (approval number: C19‐054). This study also received ethics approval (approval number: B2023‐040) from the National Center of Neurology and Psychiatry, the National Institute of Neurology and Psychiatry, which is a research sub‐center, and the Kitasato University School of Medicine and Hospital Ethics Committee in a collective review.

## Consent

All participants were fully informed about the study design and provided written consent.

## Conflicts of Interest

Ken Inada received personal fees from Daiichi‐Sankyo, Eisai, Eli Lilly, Janssen, Lundbeck Japan, Meiji Seika Pharma, Mitsubishi Tanabe Pharma, Mochida, MSD, Nipro, Novartis, Otsuka, Pfizer, Shionogi, Sumitomo Pharma, Yoshitomiyakuhin, Viatris, and he received research grant support from Mochida and Sumitomo pharma in the last 3 years. The other authors declare no conflicts of interest. The funding source had no role in the design, practice, or analysis of this study.

## Supporting information


**Table S1.** Mann–Whitney U analysis of the association of PTSD determination with fatty acid ratios, including ω3, ω6, and ω9.
**Table S2.** Mann–Whitney U analysis of the association of PTSD determination with fatty acids (%).
**Table S3.** Spearman’s correlation coefficient for blood linoleic acid levels at the time of hospitalization.

## Data Availability

We cannot provide raw data being freely available because we did not obtain agreements to release the data from the study participants and the supplier and because our ethical approval did not include the release. Instead, the data sets used and/or analyzed during this study are completely available from the corresponding author for collaborative research purposes upon reasonable request.
