# Supplementary material for: A Longitudinal Study of the Association of Blood Unsaturated Fatty Acids With Posttraumatic Stress Disorder (PTSD)
Source: Neuropsychopharmacol Rep. 2025 Feb 17;45(1):e12522. doi: 10.1002/npr2.12522 (PMC11833162; doi:10.1002/npr2.12522)
Supplement: Supplementary file 1 — Table S1. Mann–Whitney U analysis of the association of PTSD determination with fatty acid ratios, including ω3, ω6, and ω9. Table S2. Mann–Whitney U analysis of the association of PTSD determination with fatty acids (%). Table S3. Spearman’s correlation coefficient for blood linoleic acid levels at the time of hospitalization. [file NPR2-45-e12522-s001.docx]

**Supporting tables**

**Table S1**. Mann-Whitney U analysis of the association of PTSD determination with fatty acid ratios, including ω3, ω6, and ω9.

| Fatty acids Total | After 1 month | After 1 month | P-value |  |  | Fatty acids Total | After 3 months | After 3 months | P-value |
| --- | --- | --- | --- | --- | --- | --- | --- | --- | --- |
|  | PTSD judgment (-) | PTSD judgment (+) |  |  |  |  | PTSD judgment (-) | PTSD judgment (+) |  |
|  | n=32 | n=6 |  |  |  |  | n=24 | n=12 |  |
|  | Median(range) | Median(range) |  |  |  |  | median(range) | Media(range) |  |
| T/T ratio | 0（0–0.04） | 0（0–0.02) | 0.625 |  |  | T/T ratio | 0(0–0.04) | 0(0–0.03) | 0.344 |
| EPA/AA ratio | 0.215（0.04–0.88） | 0.175（0.07–0.42) | 0.861 |  |  | EPA/AA ratio | 0.23(0.05–0.68) | 0.18(0.04–0.88) | 0.655 |
| DHA/AA ratio | 0.435（0.17–1.17） | 0.37（0.24–0.79) | 0.74 |  |  | DHA/AA ratio | 0.435(0.17–1.01) | 0.46(0.22–1.17) | 0.96 |
| (EPA+DHA)/AA ratio | 0.635（0.22–2.02） | 0.585（0.35–1.2) | 0.77 |  |  | (EPA+DHA)/AA ratio | 0.675(0.22–1.69) | 0.63(0.26–2.02) | 0.96 |
| W3/W6 ratio | 0.145（0.07–0.5） | 0.145（0.11–0.24) | 0.77 |  |  | W3/W6 ratio | 0.16(0.07–0.3) | 0.145(0.08–0.5) | 0.779 |
| Total ω3 | 141.15(63–492.7) | 184.4(123.7–278.5) | 0.598 |  |  | ω3 total | 202.45（74.6–427） | 126.4（63–492.7） | 0.251 |
| Total ω6 | 1155.25(617–1824.6) | 1191.5(1065–1423.1) | 0.544 |  |  | ω6 total | 1199.2（617–1824.6） | 951.3（719–1423.1） | 0.004 |
| Total ω9 | 652.6(370.9–1685.7) | 693.4(590.2–877.2) | 0.891 |  |  | ω9 total | 774.7（404.9–1685.7） | 569.4（370.9–877.2） | 0.009 |
| Total SFA | 953.2(572.4–2191.1) | 1016.6(875.5–1164.5) | 0.77 |  |  | SFA total | 1032.75（572.4–2191.1） | 881.8（591.4–1164.5） | 0.032 |
| Total UFA | 2042.2(1170.8–3927.2) | 2176.05(1823.5–2596.1) | 0.74 |  |  | UFA total | 2252.5（1170.8–3927.2） | 1721.7（1271–2596.1） | 0.013 |
| Total MUFA | 693.8(402–1871.7) | 760.65(624–957.7) | 1 |  |  | MUFA total | 832（436.9–1871.7） | 631.4（402–957.7） | 0.016 |
| Total PUFA | 1329.2(733.9–2055.5) | 1403.85(1192.5–1638.4) | 0.571 |  |  | PUFA total | 1402.55（733.9–2055.5） | 1114.7（788.7–1638.4） | 0.02 |

T/T ratio, Thromboxane A2 to thromboxane B2 ratio, EPA/AA ratio, Eicosapentaenoic acid to arachidonic acid ratio, DHA/AA ratio, Docosahexaenoic acid to arachidonic acid ratio, (EPA+DHA)/AA ratio, Combined eicosapentaenoic and docosahexaenoic acids to arachidonic acid ratio, W3/W6 ratio, Omega-3 to omega-6 ratio, ω3, Omega-3 fatty acids, ω6, Omega-6 fatty acids, ω9, Omega-9 fatty acids, SFA, Saturated fatty acids, UFA, Unsaturated fatty acids, MUFA, Monounsaturated fatty acids, PUFA, Polyunsaturated fatty acids.

**Table S2.** Mann-Whitney U analysis of the association of PTSD determination with fatty acids (%).

| Fatty acids % weight | After 1 month | After 1 month |  |  |  | Fatty acids % weight | After 3 months | After 3 months | significant probability |
| --- | --- | --- | --- | --- | --- | --- | --- | --- | --- |
|  | PTSD judgment (-) | PTSD judgment (+) |  |  |  |  | PTSD judgment (-) | PTSD judgment (+) |  |
|  | n=32 | n=6 |  |  |  |  | n=24 | n=12 |  |
|  | median（range) | median（range) | P-value |  |  |  | median（range) | median（range) | P-value |
| Lavulinic acid(%) | 0.095（0.03–0.3） | 0.055（0.03–0.32) | 0.213 |  |  | Lavulinic acid(%) | 0.1(0.03-0.3) | 0.085(0.04-0.32) | 0.856 |
| Milestone Acid(%) | 0.75（0.46–1.87） | 0.795（0.4–1.46) | 0.77 |  |  | Milestone Acid(%) | 0.775(0.4-1.87) | 0.785(0.46-1.46) | 0.96 |
| Milestone Acid(%) | 0.04（0.02–0.17） | 0.07（0.02–0.14) | 0.356 |  |  | Milestone Acid(%) | 0.04(0.02-0.17) | 0.06(0.03-0.14) | 0.224 |
| Palmitic acid(%) | 22.135（20.52–26.48） | 21.925（20.82–24.67) | 0.77 |  |  | Palmitic acid(%) | 22.245(20.52-26.48) | 22.24(20.82-24.67) | 0.96 |
| Palmitoleic acid(%) | 1.59（0.9–3.94） | 2.055（0.95–2.25) | 0.356 |  |  | Palmitoleic acid(%) | 1.63(0.9-3.94) | 1.81(1.15-3.45) | 0.212 |
| Stialic acid(%) | 7.52（5.65–8.91） | 7.31（6.35–8.12) | 0.422 |  |  | Stialic acid(%) | 7.4(5.65-8.35) | 7.665(6.35-8.59) | 0.072 |
| Oleanic acid(%) | 21.42（17.98–27.6） | 20.62（19.12–22.24) | 0.422 |  |  | Oleanic acid(%) | 21.875(18.65-27.6) | 20.415(17.98-23.26) | 0.104 |
| Linoleic acid(%) | 28.955（19.33–34.94） | 28.19（26.52–30.49) | 0.445 |  |  | Linoleic acid(%) | 28.555(19.33-34.94) | 28.125(20.87-33.4) | 0.54 |
| G-linoleic acid(%) | 0.295（0.05–1.01） | 0.39（0.15–0.68) | 0.625 |  |  | G-linoleic acid(%) | 0.29(0.1-0.65) | 0.355(0.05-0.86) | 0.361 |
| Linoleic acid(%) | 0.765（0.35–2.08） | 0.715（0.49–0.88) | 0.445 |  |  | Linoleic acid(%) | 0.865(0.36-2.08) | 0.59(0.35-1.04) | 0.049 |
| Alakijic acid(%) | 0.25（0.14–0.35） | 0.265（0.21–0.29) | 0.571 |  |  | Alakijic acid(%) | 0.24(0.14-0.31) | 0.265(0.19-0.35) | 0.436 |
| Ecosanic acid(%) | 0.16（0.1–0.27） | 0.135（0.11–0.18) | 0.199 |  |  | Ecosanic acid(%) | 0.16(0.1-0.25) | 0.14(0.11-0.27) | 0.177 |
| Eicosazione(%) | 0.21（0.15–0.26） | 0.21（0.2–0.25) | 0.77 |  |  | Eicosazione(%) | 0.215(0.15-0.26) | 0.215(0.18-0.26) | 0.753 |
| 5-8-11 Ecosatriatic acid(%) | 0.07（0.03–0.35） | 0.095（0.07–0.13) | 0.147 |  |  | 5-8-11 Ecosatriatic acid(%) | 0.07(0.03-0.15) | 0.11(0.03-0.35) | 0.156 |
| Jihomo-G-linolenic acid(%) | 1.125（0.62–2.15） | 1.345（1.13–1.89) | 0.041 |  |  | Jihomo-G-linolenic acid(%) | 1.13(0.78-1.64) | 1.44(0.62-2.15) | 0.104 |
| Alacridic acid(%) | 6.165（3.68–10.33） | 7.845（5.63–9.82) | 0.062 |  |  | Alacridic acid(%) | 6.225(3.68-9.35) | 6.345(5.24-10.33) | 0.251 |
| Ecosapentanoic acid(%) | 1.22（0.27–5.13） | 1.295（0.64–2.36) | 0.711 |  |  | Ecosapentanoic acid(%) | 1.4(0.27-2.93) | 1.275(0.41-5.13) | 0.804 |
| Vegetarian acid(%) | 0.605（0.31–0.86） | 0.635（0.52–0.7) | 0.83 |  |  | Vegetarian acid(%) | 0.58(0.31-0.78) | 0.635(0.42-0.86) | 0.237 |
| Elsinic acid(%) | 0（0–0.08） | 0（0–0.04) | 0.377 |  |  | Elsinic acid(%) | 0(0-0.06) | 0(0-0.08) | 0.908 |
| Decosatetraenic acid(%) | 0.165（0.08–0.31） | 0.185（0.12–0.22) | 0.544 |  |  | Decosatetraenic acid(%) | 0.16(0.08-0.27) | 0.18(0.11-0.31) | 0.456 |
| Decosapentane(%) | 0.49（0.21–1.08） | 0.56（0.45–0.66) | 0.469 |  |  | Decosapentane(%) | 0.5(0.28-0.84) | 0.495(0.21-1.08) | 0.96 |
| Regnoselenic acid(%) | 0.605（0.31–0.79） | 0.605（0.5–0.69) | 0.83 |  |  | Regnoselenic acid(%) | 0.57(0.31-0.79) | 0.63(0.4-0.72) | 0.067 |
| Dekosaponinic acid(%) | 2.67（0.98–7.08） | 2.9（2.31–4.42) | 0.625 |  |  | Dekosaponinic acid(%) | 3.035(0.98-4.45) | 2.74(1.85-7.08) | 0.908 |
| Neuronic acid(%) | 1.125（0.56-1.66） | 1.23（1.02-1.33) | 0.711 |  |  | Neuronic acid(%) | 1.07(0.56-1.61) | 1.28(1.01-1.66) | 0.049 |

**Table S3.** Spearman's correlation coefficient for blood linoleic acid levels at the time of hospitalization

|  | **Degree** | **Correlation coefficient** | **Two-tailed P-value** |
| --- | --- | --- | --- |
| Total cholesterol | 31 | 0.676 | ＜0.001 |
| LDL-Cholesterol | 25 | 0.745 | ＜0.001 |
| Serum cholinesterase | 22 | 0.482 | 0.023 |
| Neutral Fat | 28 | 0.694 | ＜0.001 |
| FIB | 32 | 0.409 | 0.02 |
| PT-percentage | 32 | 0.555 | ＜0.001 |
| PT-T | 32 | -0.567 | ＜0.001 |
| CRP levels | 32 | 0.362 | 0.042 |
| White Blood Cell Count | 32 | -0.507 | 0.003 |
| Neutrophil count | 32 | -0.45 | 0.01 |
| Monocytes | 32 | 0.357 | 0.045 |

LDL-Cholesterol, Low-density lipoprotein cholesterol; FIB, Fibrinogen; PT-percentage, Prothrombin percentage, PT-T, Prothrombin Time; CRP levels, C-reactive protein levels.
